# Prevalence and Risk Factors of Post-traumatic Stress Disorder Symptoms in Students Aged 8–18 in Wuhan, China 6 Months After the Control of COVID-19

**DOI:** 10.3389/fpsyg.2021.740575

**Published:** 2021-10-13

**Authors:** Yue Chen, Zhuohong Zhu, Fei Lei, Shulan Lei, Jing Chen

**Affiliations:** ^1^Key Laboratory of Mental Health, Institute of Psychology, Chinese Academy of Sciences, Beijing, China; ^2^Department of Psychology, University of Chinese Academy of Sciences, Beijing, China; ^3^Key Laboratory of Behavioral Science, Chinese Academy of Sciences, Institute of Psychology, Beijing, China

**Keywords:** adolescents, COVID-19, post-traumatic stress disorder, psychological inflexibility, mental health

## Abstract

**Objectives:** To explore the prevalence of post-traumatic stress disorder (PTSD) symptoms and the factors influencing mental health symptoms in students aged 8–18 in Wuhan, China at 6 months after the COVID-19 pandemic was controlled.

**Methods:** Questionnaires were distributed to students aged 8–18 in Wuhan through an online platform from September to October 2020, and 15,993 valid surveys were returned, resulting in a response rate of 75.4%. The data related to symptoms of PTSD, anxiety, depression, stress and psychological inflexibility levels, as well as demographic information about the population. Hierarchical multiple regression analyses were performed to examine the predictive effects.

**Results:** In total, 11.5% of the students met the criteria for clinically concerning PTSD symptoms. Psychological inflexibility was associated with PTSD symptoms, depression, anxiety, and stress symptoms (β = 0.45, 0.63, 0.65 and 0.69, respectively, with ΔR^2^ = 0.16, 0.32, 0.34 and 0.39, respectively, *p* < 0.001) in children and adolescents.

**Conclusion:** This study investigated the impacts of COVID-19 on the mental health status among students aged 8–18 in Wuhan. Even at 6 months after the outbreak was brought under control, some students were still affected. Psychological inflexibility was correlated with psychological symptoms in students. Therefore, methods to reduce psychological inflexibility may help improve the mental health states of students as part of psychological interventions.

## Introduction

The recent novel coronavirus disease (COVID-19) outbreak is characterized by rapid transmission and wide infection range, presenting great difficulty for effective control. As of 5:08 pm. CEST, July 12th, 2021, COVID-19 had spread to 223 countries, with 186,638,285 people infected and 4,035,037 related death [World Health Organization (WHO), 2021]. This public health emergency has not only endangered people’s physical health, but also has had a significant impact on people’s mental health (Liang et al., 2020). To limit the spread of the virus, the government advised people to reduce their social activities and maintain social distance, which can increase isolation, loneliness, stress, and anxiety [Centers for Disease Control and Prevention (CDC), 2020]. In the early days of the outbreak, the rapidly increasing number of cases daily and the lack of knowledge about the virus led to psychological stress (Cheng et al., 2020). People experience a range of negative emotions when faced with a new and deadly infectious disease that requires mandatory isolation (Braun-Lewensohn, 2015). COVID-19 cases were first reported in Wuhan, and the city was quickly shut down to limit spread. On April 23, 2020, COVID-19 was brought under control in Wuhan, so the city lifted its blockade and recovery began. However, the long-term psychological impacts of this epidemic and the scope of these impacts have not been determined. It is important to assess these impacts to assess the mental health of the local people in Wuhan, to guide mental health intervention, and to provide theoretical support for study of the mental health of people in other parts of the world affected by the pandemic.

The deadly nature of COVID-19 and its very high infection rate can induce post-traumatic stress disorder (PTSD) in people (Liang et al., 2020). PTSD is a condition caused by an individual experiencing or witnessing traumatic events such as combat, crime, accidents, or a natural disaster, that exceed the limits of an individual’s psychological capacity (American Psychiatric Publishing, 2013). Potentially traumatic events such as acute illness and severe hospitalization can also cause PTSD (Tarsitani et al., 2021), and perceived threats to life and fear of death can play key roles in the development of PTSD (Heir et al., 2016). PTSD seriously decreases psychological and social function, adversely affecting life quality. PTSD has four core symptoms: repetition of traumatic experiences, constant avoidance of stimuli associated with traumatic events, negative cognitive and emotional changes, and increased alertness (American Psychiatric Publishing, 2013). A study that analyzed data from 26 population surveys from the World Health Organization’s World Mental Health Survey determined that among people exposed to trauma, the lifetime prevalence of PTSD was 5.6% in adults and approximately 16% in children and adolescents (Koenen et al., 2017; Kolaitis, 2017). The outbreak of Severe Acute Respiratory Syndrome (SARS) in the early 20th century was similar to the current situation. It is also a highly contagious respiratory infection, and studies of long-term mental illness in SARS survivors have shown that post-traumatic stress disorder (PTSD) is the most common resulting long-term mental illness (Mak et al., 2009). A widespread prevalence of PTSD related to the COVID-19 outbreak has already been reported. One month after the outbreak, PTSD among health-care workers in China was 3.8% (Yin et al., 2020), with a 12.8% prevalence of PTSD symptoms among people aged 14–35 in China (Liang et al., 2020). Children and adolescents lack effective coping skills to adapt to, and recover from, traumatic experiences, making them more susceptible than adults to PTSD (Braun-Lewensohn, 2015). In addition, COVID-19 presents a mortal risk, and the fear of injury and death in unexpected and unprepared circumstances can result in panic, fear and tension (Xu et al., 2016). Two weeks after the start of the COVID-19 outbreak in China, a general population mental health survey showed that approximately one-third of the participants were experiencing moderate to severe anxiety (Wang et al., 2020a). A meta-analysis of studies conducted on health-care workers after the outbreak showed the prevalence rates of anxiety and depression were 23.2% and 22.8%, respectively (Pappa et al., 2020). However, there have been limited studies on the mental health states of children and adolescents in Wuhan.

Many factors affect the level of mental health, including gender, age (Qi et al., 2020), education level, and income level (Yue et al., 2020). Psychological factors also affect psychological symptoms, including psychological support (Rezayat et al., 2020), intrusive rumination (Qi et al., 2020), negative coping styles (Liang et al., 2020), resilience (Chi et al., 2020) and psychological inflexibility (Marx and Sloan, 2005). Psychological inflexibility refers to an individual’s efforts to avoid unwanted emotional, cognitive and physical feelings at the expense of more effective and value-driven actions (Bond et al., 2011; Levin et al., 2013; DeBeer et al., 2018). The inability of a person to effectively change their behavior in response to immediate stress or changing environmental requirements may exacerbate stress and potentially contribute to the development, maintenance, and worsening of a wide range of psychological problems, such as depression, anxiety, and eating disorders (Kashdan and Rottenberg, 2010; Levin et al., 2013; Stange et al., 2017), Previous studies have shown that psychological inflexibility can predict PTSD symptoms in war veterans (Marx and Sloan, 2005), women who have experienced school shootings (Kumpula et al., 2011), and inpatient adolescents (Schramm et al., 2020). After a traumatic event, painful thoughts, feelings (including feelings of guilt and shame), and associated physical reactions can occur upon exposure to reminders associated with the traumatic event. When this happens, high psychological inflexibility individuals may try to suppress and avoid these thoughts and feelings by distraction or other avoidance tactics, which in turn may increase the risk of developing PTSD later in life (Norman et al., 2014; Kachadourian et al., 2021). However, whether psychological inflexibility is related to mental health after COVID-19 has not been explored.

Most recent studies have focused on the mental health status of adults with COVID-19, with little study of children and adolescents. Therefore, the goals of this cross-sectional study were to understand the incidence of PTSD symptoms in students aged 8–18 in Wuhan 6 months after COVID-19 was controlled, and to explore the relation between psychological inflexibility and students’ mental health.

## Materials and Methods

### Study Participants

We surveyed students aged 8–18 (fourth grade of primary school through third grade of high school) in Wuhan from 16th September to 9th October 2020, approximately half a year after COVID-19 was brought under control in Wuhan. Questionnaires were distributed through an online platform. Before the test began, students and their parents were told the purpose of the measurements and read the informed consent to understand that they could refuse to answer or quit answering at any time. A total of 21,218 questionnaires were collected. An indicator question in the questionnaire, which was “Please select the last option for this question,” was included. Participants who consistently chose one option and those who answered the indicator incorrectly were considered as not being serious and, therefore, were excluded. Data on 15,993 students remained for analysis (75.4%). The average age of the participants was 12.26 (SD = 2.14), and the detailed demographic information is shown in Table 1.

**TABLE 1:** Characteristics of study participants (*N* = 15,993).

Demographics		Samples	Percentage
Gender	Male	8,100	50.6%
	Female	7,893	49.4%
Education	Primary school	8,188	51.2%
	Middle school	5,440	34.0%
	High school	2,365	14.8%
Only child	Yes	9,380	58.7%
	No	6,613	41.3%
Infected with COVID-19	Yes	30	0.2%
	No	15,963	99.8%
Perceived family atmosphere	Warm	12,264	76.7%
	Between warm and not warm	3,407	21.3%
	Not warm	322	2.0%
Perceived family economic level	Poor	420	2.6%
	Fair	14,394	90.0%
	Rich	1,179	7.4%
Personal physical health status	Very good	9,646	60.3%
	Good	4,310	26.9%
	General	1,496	9.4%
	Poor health (frequent illness)	226	1.4%
	Poor health (always illness)	20	0.1%
	Currently suffering from a certain disease	295	1.8%
Acquaintance with people who were infected	Yes, know people who died because of COVID-19	353	2.2%
	Yes, know people who were infected by COVID-19	668	4.2%
	No	14,972	93.6%

### Measurements

#### The Children’s Revised Impact of Event Scale

The Children’s Revised Impact of Event Scale (CRIES-13, Lau et al., 2013) was used to assess PTSD symptoms. This scale is often used in studies on adolescents, and it has shown good reliability and validity in China (Wang et al., 2020b; Xu et al., 2021). The 13-item questionnaire allowed for easy administration to children, and it measured three representative symptoms of PTSD: intrusion, avoidance and hyperarousal. This is a 4-point scale (0 = none, 1 = rarely, 3 = sometimes, 5 = often). The higher the score, the more severe the condition. If the total scale score of the subject is equal to, or higher than, 30 points, then the subject is considered likely to develop PTSD. The scale is designed specifically to screen and assess the severity of PTSD in children and adolescents, and is not a formal diagnosis. In this study, the items of the questionnaire were adjusted to address impacts of the COVID-19 outbreak (e.g., we changed “Did you try to remove it from your memory” to “Did you try to remove the COVID-19 epidemic from your memory?”). Cronbach’s α coefficient of the whole questionnaire was 0.85, and intrusion, avoidance, and hyperarousal had Cronbach’s α coefficients of 0.83, 0.78, and 0.68, respectively.

#### The Chinese Short Version of Depression Anxiety and Stress Scale

The Depression Anxiety and Stress Scale (DASS-C21, Wang et al., 2016) was used to measure participants’ perceived severity of symptoms related to depression, anxiety and stress. The scale includes three subscales of depression, anxiety and stress, and each subscale contains seven items, for a total of 21 items. The scale adopts Likert’s five-point scoring method, from 1 (completely inconsistent) to 5 (completely consistent). The scale is used to assess an individual’s negative emotional level over the past week, and the higher the score, the more severe the negative emotional level. The questionnaire has good reliability and validity among Chinese teenagers (Zhang et al., 2016). In this study, the Cronbach’s α coefficients for the depression, anxiety, and stress subscales of DASS-C21 and that for the entire scale were 0.90, 0.87, 0.89, and 0.96, respectively.

#### The Avoidance and Fusion Questionnaire for Youth

The Avoidance and Fusion Questionnaire for Youth (AFQ-Y8, Chen et al., 2019) was used to measure psychological inflexibility in children and adolescents. There are eight items in the questionnaire, and responses to each item on the AFQ-Y8 were recorded on a 5-point Likert scale ranging from 1 (completely inconsistent) to 5 (completely consistent). Response scores were summed, with higher scores indicating a higher level of psychological inflexibility. The Cronbach’s α coefficient of the global score was 0.91 in this study.

### Statistical Analyses

SPSS 26.0 software was used for data analysis. Means and standard deviations (SD) were calculated to describe the scale scores. Cronbach’s α coefficient was used to represent internal consistency of CRIES-13, DASS-C21, and AFQ-Y8. Pearson correlation coefficients were calculated to describe the correlations between variables. Using the two category variables of gender and only child or not, the relationships between psychological inflexibility and psychological symptoms were explored using a hierarchical multiple regression analysis. Model 1 included demographic factors. In Model 2, the psychological inflexibility total score was added to the regression equation. Durbin-Watson (DW) was used to calculate the independence of the residuals of the dependent variables, and variance inflation factor (VIF) was used to measure the multicollinearity of the independent variables. A value of *p* < 0.05 (two-tailed) was considered statistically significant and a value of VIF < 5 was considered free of multicollinearity.

## Results

### Mental Health Status and Prevalence of Post-traumatic Stress Disorder

The average CRIES-13 score was 15.91 ± 11.14, with scores of 5.5 (SD = 4.51) for intrusion, 4.34 (SD = 4.53) for avoidance, and 6.06 (SD = 4.76) for hyperarousal. A total of 840 (11.5%) CRIES-13 scores were equal or higher than 30 points, the standard of clinical concern. The average scores of AFQ-Y8 and depression, anxiety, and stress dimensions in DASS-C21 were 34.42 ± 7.01, 9.65 ± 4.84, 9.59 ± 4.54, and 10.74 ± 5.39, respectively.

### Associations Among Post-traumatic Stress Disorder, Mental Health, and Psychological Inflexibility

Correlation analysis results are presented in Table 2, and show a significant negative correlation between psychological inflexibility and PTSD and between psychological inflexibility and mental health (all *p*-values less than 0.01).

**TABLE 2:** Correlations among PTSD, mental health, and psychological inflexibility.

	1	2	3	4	5
**1. PTSD**	–				
**2. Depression**	0.40**	–			
**3. Anxiety**	0.45**	0.83**	–		
**4. Stress**	0.50**	0.85**	0.86**	–	
**5. Psychological inflexibility**	0.47**	0.72**	0.72**	0.76**	–

*^∗∗^p-value < 0.01.*

A hierarchical multiple regression analysis of the CRIES-13 data showed that DW = 1.93, indicating that residual values of dependent variables were independent and lacked autocorrelation. PTSD symptoms measured by the CRIES-13 total score were regressed on demographic variables, with a 9% co-explanation variation for demographic factors. Gender, only child or not, age, perceived family atmosphere, perceived family economic level, personal physical health status and knowing people who were infected, were all found to be significantly associated with PTSD symptoms (Model 1, Table 3). After adding psychological inflexibility, Model 2 (Table 3) showed that the overall explanatory variation increased by 16%, indicating that psychological inflexibility contributed to PTSD symptoms after controlling for demographic factors. More severe psychological inflexibility levels correlated with more severe PTSD symptoms.

**TABLE 3:** Hierarchical multiple regression analyses using the CRIES-13 total score for adolescents as the outcome variable.

			Model 1 Demographic characteristics				Model 2 Psychological inflexibility			
		Intramodal variables	B	β	*t*	VIF	B	β	*t*	VIF
**Independent variables**	**1**	Male and Female	−0.60	−0.03	−3.57***	1.01	−0.90	−0.04	−5.87***	1.01
		Only child and Non-only child	0.96	0.04	5.58***	1.02	0.69	0.03	4.44***	1.02
		Age	1.04	0.20	25.88***	1.04	0.82	0.16	22.53***	1.05
		Perceived family atmosphere	2.80	0.12	15.23***	1.10	−0.53	−0.02	−2.98**	1.23
		Perceived family economic level	−2.17	−0.06	−7.99***	1.02	−1.22	−0.03	−4.92***	1.03
		Personal physical health status	0.94	0.08	10.24***	1.07	0.14	0.01	1.62	1.10
		Knowing people who were infected	−1.25	−0.04	−5.17***	1.01	−0.65	−0.02	−2.98**	1.02
		
	**2**	**Psychological inflexibility**					0.71	0.45	58.44***	1.24

**Model summary**	***R* ^2^**		0.09				0.25			
	***F***		221.66				662.34			
	***P***		0.000				0.000			
	**ΔR^2^**		0.09				0.16			
	**ΔF**		221.66				3415.63			
	**Δ*P***		0.000				0.000			

*^∗∗^p-value < 0.01. ^∗∗∗^p-value < 0.001.*

The DW values of the regression models of depression, anxiety, and stress were 1.98, 1.97, and 2.01, respectively, indicating that the residuals of the dependent variables were independent of each other and lacked autocorrelation. Table 4 shows the regression analysis results of mental health status as the dependent variable. Model 1 shows that the results measured in each dimension of DASS-21 are regressed on the demographic variables. Demographic factors accounted for 25.0, 20.0, and 22.0% of depression, anxiety, and stress, respectively. Gender, only child or not, age, perceived family atmosphere, perceived family economic level, personal physical health status and knowing people who were infected are all factors related to overall mental health status. With the inclusion of psychological inflexibility, Model 2 showed that the overall explanatory variations of depression, anxiety and stress increased by 31.0, 34.0, and 39.0%, respectively, indicating that psychological inflexibility was associated with mental health status. The higher the psychological inflexibility, the more serious the manifestations of depression, anxiety and stress.

**TABLE 4:** Hierarchical multiple regression analysis using the mental health score for adolescents as the outcome variable.

																										
			Depression								Anxiety								Stress							
			Model 1				Model 2				Model 1				Model 2				Model 1				Model 2			
			Demographic characteristics				Psychological inflexibility				Demographic characteristics				Psychological inflexibility				Demographic characteristics				Psychological inflexibility			
		Intramodal variables	B	β	*t*	VIF	B	β	*t*	VIF	B	β	*t*	VIF	B	β	*t*	VIF	B	β	*t*	VIF	B	β	*t*	VIF
																										
Independent	1	Male and Female	0.14	0.01	9.28***	1.01	1.60	–0.01	–1.05	1.01	0.28	0.03	4.39***	1.01	0.11	0.01	2.19*	1.01	0.19	0.02	2.53*	1.01	–0.03	–0.00	–0.60	1.01
variables		Only child and Non-only child	0.25	0.03	2.06*	1.02	–0.06	0.01	1.64	1.02	0.32	0.03	4.83***	1.02	0.16	0.02	3.20**	1.02	0.21	0.02	2.75**	1.02	0.01	0.00	0.23	1.02
		Age	0.16	0.07	3.64***	1.04	0.08	0.01	2.48*	1.05	0.17	0.08	11.39***	1.04	0.05	0.02	4.11***	1.05	0.24	0.10	13.59***	1.04	0.08	0.03	6.50***	1.05
		Perceived family atmosphere	4.04	0.40	9.68***	1.10	0.03	0.20	34.34***	1.23	3.10	0.33	44.12***	1.10	1.14	0.12	20.10***	1.23	4.01	0.36	48.77***	1.10	1.51	0.14	24.56***	1.23
		Perceived family economic level	–0.97	–0.06	55.57***	1.02	2.01	–0.03	−4.72***	1.03	–0.84	–0.06	−8.13***	1.02	–0.28	–0.02	−3.53***	1.03	–1.01	–0.06	−8.35***	1.02	–0.29	–0.02	−3.44**	1.03
		Personal physical health status	0.77	0.15	−9.03***	1.07	–0.39	0.06	10.12***	1.10	0.83	0.17	23.76***	1.07	0.36	0.08	13.24***	1.10	0.95	0.17	23.19***	1.07	0.35	0.06	11.80***	1.10
		Knowing people who were infected	–0.47	–0.03	21.33***	1.01	0.28	–0.01	–1.44	1.02	–0.50	–0.04	−5.36***	1.01	–0.14	–0.01	−2.04*	1.02	–0.58	–0.04	−5.40***	1.01	–0.14	–0.01	–1.78	1.02

	2	Psychological inflexibility					–0.11	0.63	107.65***	1.24					0.42	0.65	107.82***	1.24					0.53	0.69	125.35***	1.24

																										
Model summary	*R* ^2^		0.25				0.56				0.20				0.54				0.22				0.61			
	*F*		745.43				2573.50				575.12				2322.33				658.73				3107.18			
	*P*		0.000				0.000				0.000				0.000				**0.000**				**0.000**			
	ΔR^2^		0.25				0.31				0.20				0.34				0.22				0.39			
	ΔF		745.43				11587.69				575.12				11625.22				658.73				15713.75			
	Δ*P*		0.000				0.000				0.000				0.000				**0.000**				**0.000**			

*^∗^p-value < 0.05. ^∗∗^p-value < 0.01. ^∗∗∗^p-value < 0.001.*

## Discussion

This is the first study to explore the possible effects of COVID-19 on the mental health of students in Wuhan. The results showed that approximately half a year after COVID-19 was controlled, 11.5% of students aged 8–18 in Wuhan had clinically concerning PTSD symptoms caused by the epidemic. This result differs somewhat from previous research. Results of a meta-analysis of 39 studies showed that the prevalence of PTSD in children and adolescents 1, 2, 3, and 4 months after earthquake and flood disasters was 19.2, 30.0, 24.4, and 20.4%, respectively (Rezayat et al., 2020). Three years after the Lushan earthquake, the prevalence of PTSD among adolescents was 19.6% (Wang et al., 2020b). Chi et al. (2020) studied Chinese college students a month after the COVID-19 outbreak and found a 30.8% prevalence of PTSD symptoms. These percentages are higher than the percentages reported here, and may reflect differences in the tools and screening criteria used and the age of the participants. The different PTSD incidence rates may also be influenced by the varied time of measurement after the traumatic event, as the prevalence of PTSD is reduced by approximately 50% 3 months after a traumatic event, with moderate reduction of symptoms (Hiller et al., 2016). This study was conducted approximately 6 months after COVID-19 was controlled in Wuhan; consequently, many students may have already recovered from PTSD symptoms. In addition, the residents of Wuhan received attention and medical help from the whole country, and children likely received more support and attention from parents and schools than college students. Finally, the type of traumatic event may also contribute to differences in the incidence level of PTSD symptoms, because natural disasters, such as earthquakes and floods, cause more property damage than public health emergencies. Therefore, the aftermath of a natural disaster may be more visually apparent to those who experienced it. However, during the COVID-19 pandemic, a lack of understanding of the virus, as well as limited behavioral and interpersonal communications, created an invisible climate of terror.

This study revealed a significant correlation between PTSD symptoms caused by COVID-19 and adolescent depression, anxiety and stress levels, which is consistent with previous research. Early studies showed that depression is a factor that predicts PTSD symptoms (Giaconia et al., 1995; North et al., 1997). Subsequent studies found a significant association between PTSD symptoms and depression in adolescents (Watson, 2005; Fan et al., 2015). The city was locked down and personal movements were restricted, which may have caused psychological stress for students. In addition, daily exposure to relevant media coverage, restrictions on going out, unknown viral characteristics and the fear of being infected, may be stimuli for students, causing stress and trauma, which could trigger other psychological symptoms. Future longitudinal studies may explore the causal relationship between PTSD symptoms and adolescent mental health. In addition, the results of the hierarchical multiple regression analysis showed that after controlling for demographic factors, psychological inflexibility may be a common factor that related to PTSD, depression, anxiety and stress symptoms. This result is consistent with a previous study by Levin et al. (2013) of 972 college students, which found that psychological inflexibility is associated with comorbidities of depression, anxiety, and substance use disorders. This is also consistent with the process-based therapy (PBT) hypothesis, proposed by Hofmann and Hayes (2019), that comorbidities and patient heterogeneity are common in the psychosocial disorder group, making it difficult to diagnose a single disease by diagnostic criteria. Therefore, new clinical interventions should focus on process-based therapies, which emphasize the process of change. PBT advocates that the same psychological processes may be behind different psychological symptoms, so addressing the core psychological processes during treatment is required to relieve various diseases and promote health (Dindo et al., 2017).

In addition to psychological processes, such as psychological inflexibility, some demographic factors may also contribute to psychological symptoms. Age, perceived family atmosphere and family economic level may be predictors of four types of mental health disorders in adolescents. It is difficult to improve these factors through clinical interventions, but they allow us to identify at-risk children and may help involve adolescents’ parents into the process of psychological intervention, thereby comprehensively promoting the mental health of adolescents. As the first city to report COVID-19, the mental health status of Wuhan’s residents is of great significance for follow-up studies on psychological effects related to the epidemic. Future research may further explore the causal relationship between psychological inflexibility and psychological symptoms, as well as the significance of this relationship for clinical intervention.

This study had the following limitations: First, 8–18 years old were studied, and the results may differ among smaller age groups. Future research should explore the mental health characteristics of students at different stages of education. Second, this study only collected the cross-sectional data of a large sample. Future studies should collect longitudinal data to better understand the development and interactions of mental health problems in adolescents after traumatic events, as well as the role of psychological inflexibility in this process. In addition, this study used a self-report instrument, which could make the results less clinically accurate. A more objective approach to assessing the mental health of participants may be used in the future. Finally, all the subjects in this study were from Wuhan; consequently, the measured results may not be representative of all populations. Future research should focus on the characteristics, developmental trajectory and influencing factors of PTSD and mental health in adolescents on a wider geographic scale.

## Conclusion

This study found that 6 months after the control of COVID-19, the prevalence of PTSD symptoms among adolescents in Wuhan was 11.5%. PTSD symptoms caused by the epidemic were significantly related to the adolescents’ current mental health states. Psychological inflexibility was associated with mental health disorder symptoms. This is the first study to investigate the prevalence of PTSD symptoms, and their relationship with psychological inflexibility, in children and adolescents. The results of this study have important implications for future research and planning for emerging diseases. They will help public health and educational systems better address the impacts of COVID-19, or similar disasters, on adolescent mental health.

## Data Availability Statement

The raw data supporting the conclusions of this article will be made available by the authors, without undue reservation.

## Ethics Statement

The studies involving human participants were reviewed and approved by the Research Ethics Committee, Institute of Psychology, CAS. Written informed consent to participate in this study was provided by the participants’ legal guardian/next of kin.

## Author Contributions

YC and ZZ made the main contributions to this study including the concept, and writing and revising the manuscript. FL and SL provided data analysis strategies for this study. JC provided suggestions on revisions and confirmed the final version to be published. All authors contributed to the article and approved the submitted version.

## Conflict of Interest

The authors declare that the research was conducted in the absence of any commercial or financial relationships that could be construed as a potential conflict of interest.

## Publisher’s Note

All claims expressed in this article are solely those of the authors and do not necessarily represent those of their affiliated organizations, or those of the publisher, the editors and the reviewers. Any product that may be evaluated in this article, or claim that may be made by its manufacturer, is not guaranteed or endorsed by the publisher.
